# Energy Efficient Memristor Based on Green‐Synthesized 2D Carbonyl‐Decorated Organic Polymer and Application in Image Denoising and Edge Detection: Toward Sustainable AI

**DOI:** 10.1002/advs.202408648

**Published:** 2024-09-09

**Authors:** Pratibha Pal, Hanrui Li, Ruba Al‐Ajeil, Abdul Khayum Mohammed, Ayman Rezk, Georgian Melinte, Ammar Nayfeh, Dinesh Shetty, Nazek El‐Atab

**Affiliations:** ^1^ Smart, Advanced Memory Devices and Applications (SAMA) Laboratory Electrical and Computer Engineering Program Computer Electrical Mathematical Science and Engineering Division King Abdullah University of Science and Technology (KAUST) Thuwal 23955 Kingdom of Saudi Arabia; ^2^ Department of Chemistry Khalifa University of Science & Technology Abu Dhabi 127788 UAE; ^3^ Department of Electrical Engineering Khalifa University of Science & Technology Abu Dhabi 127788 UAE; ^4^ Core Labs King Abdullah University of Science and Technology Thuwal 23955‐6900 Saudi Arabia; ^5^ Center for Catalysis & Separations (CeCaS) Khalifa University of Science & Technology Abu Dhabi 127788 UAE

**Keywords:** 2D polymers, green synthesis, memristor, neuromorphic computing, sustainable electronics

## Abstract

According to the United Nations, around 53 million metric tons of electronic waste is produced every year, worldwide, the big majority of which goes unprocessed. With the rapid advances in AI technologies and adoption of smart gadgets, the demand for powerful logic and memory chips is expected to boom. Therefore, the development of green electronics is crucial to minimizing the impact of the alarmingly increasing e‐waste. Here, it is shown the application of a green synthesized, chemically stable, carbonyl‐decorated 2D organic, and biocompatible polymer as an active layer in a memristor device, sandwiched between potentially fully recyclable electrodes. The 2D polymer's ultramicro channels, decorated with C═O and O*─*H groups, efficiently promote the formation of copper nanofilaments. As a result, the device shows excellent bipolar resistive switching behavior with the potential to mimic synaptic plasticity. A large resistive switching window (10^3^), low SET/RESET voltage of ≈0.5/−1.5 V), excellent device‐to‐device stability and synaptic features are demonstrated. Leveraging the device's synaptic characteristics, its applications in image denoising and edge detection is examined. The results show a reduction in power consumption by a factor of 10^3^ compared to a traditional Tesla P40 graphics processing unit, indicating great promise for future sustainable AI‐based applications.

## Graphical abstract

A memristor based on a green‐synthesized, chemically stable, biocompatible 2D organic polymer along with potentially recyclable electrodes is demonstrated. This device exhibits excellent resistive switching, stable synaptic features, showing its promising application in image denoising and edge detection. The results confirm the device's higher energy‐efficiency compared to traditional GPUs, indicating great promise for future sustainable AI‐based applications.

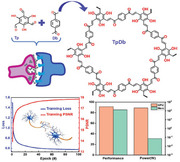

## 1 Introduction

The explosion of information technology demands efficient storage and processing of massive data sets. Traditional von‐Neumann architectures, with their separate memory and processor units, are slow and power‐hungry, hindering Big Data processing. Multifunctional memory devices offer a promising solution, enabling simultaneous data storage and processing.^[^ ^1^, ^2^, ^3^, ^4^, ^5^ ^]^ Notably, memristor, a bio‐inspired memory device, was developed as an efficient technology for in‐memory computing similar to the brain, also known as neuromorphic computing. A memristor is a two‐terminal device with a resistive switching nature, which can regulate and remember the flow of electrons in an electrical circuit that can be controlled through the formation and rupture of a conductive filament upon the application of an electric potential in between the electrodes.^[^ ^6^, ^7^, ^8^, ^9^, ^10^, ^11^, ^12^ ^]^ Remarkably, both organic and inorganic‐based memristors were developed with exciting features. Compared to inorganic materials, organic materials have advantages including low cost, flexibility, and solution processability. Meanwhile, the design of an effective organic memristor requires solid‐state redox activity for continuous regulation of the conductivity of the device. Recently, two dimensional (2D) redox‐active polymers were explored as effective organic memristors. However, most of them suffer poor chemical stability that affects the long‐term performance of memristor devices. In addition, the availability of nanochannels decorated with suitable metal‐anchoring groups serves organic 2D‐polymers as an excellent platform for the formation of metal nanofilaments. Besides, it is advised to develop scalable and green organic polymers for memristor applications considering the minimization of electronic waste, in alignment with the UN sustainability development goals. In general, there are several approaches to promoting sustainable electronics, including the use of biodegradable, recyclable, nontoxic, repairable, or energy‐efficient devices and fabrication processes.^[^ ^13^, ^14^, ^15^, ^16^, ^17^, ^18^ ^]^ While “green”/“sustainable” can have varying degrees in research, with some devices being fully biodegradable or recyclable and others incorporating these features with less eco‐friendly materials, as long as the design contributes to a lessened environmental footprint, it has still been considered and referred to as “green electronics.”

Keeping all these in perspective, we used a chemically stable 2D‐organic polymer equipped with carbonyl group (C═O), and comprising of sp^2^ carbon‐connections, as an active layer in a memristor. We have mechanochemically synthesized the 2D polymer (TpDb) through an aldol condensation‐based reaction of Tp (*1, 3, 5*‐ triformylphloroglucinol) and Db *1, 4*‐diacetylbenzene without using any organic solvents. The TpDb exhibits ultramicro channels decorated with C═O and O*─*H functional groups which aid in the formation of metal (herein Cu) nano filaments efficiently. Powder X‐ray diffraction (PXRD) analysis of TpDb revealed a broad peak at 2θ ≈8.0° and an amorphous range at 2θ ≈15° to 30°, indicating ABC stacking of 2D layers with semiperiodic nature. This ABC stacking likely originates from interlayer hydrogen bonding, resulting in a 2D framework with ultramicrochannels (Figure S1, Supporting Information). The presence of electronegative functional groups and ultramicrochannels facilitates efficient metal ion diffusion and electrochemical metallization (Figure S2, Supporting Information). Thus, the formation or rupturing of a metallic conductive filament (CF) under the application of an electric field results in resistive switching features. Remarkably, the fabricated memristor device showed the ability to mimic synaptic plasticity. Copper (Cu) and platinum (Pt) were chosen as electrodes as they can be ideally fully recycled. More specifically, the Cu/TpDb/Pt device can imitate synaptic behaviors, such as short‐term plasticity (STP), long‐term potentiation (LTP), long‐term depression (LTD), and paired‐pulse facilitation (PPF), all of which are highly desired features for the artificial intelligence (AI)‐based applications in noise reduction and edge detection. The good electronic properties, environmentally friendly nature, and ingenious structure render the TpDb a suitable candidate for neuromorphic computing. More importantly, the combination of a green synthesized and biocompatible switching layer with electrodes that can be potentially fully recycled, in addition to the high energy efficiency, can contribute to sustainable electronics and minimize the impact of e‐waste.

## 2 Results and Discussion

**Figure 1:**
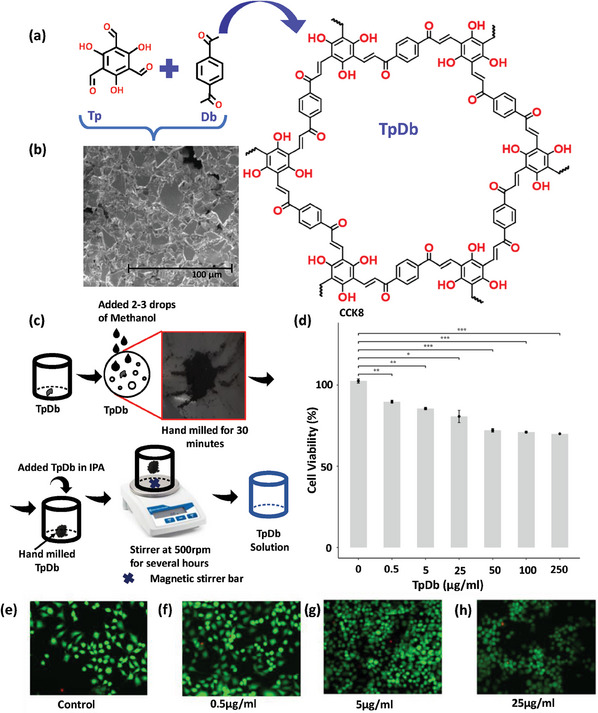
a) The schematic representation of the synthesis of TpDb. b) The SEM image of TpDb top view. c) The schematic of preparation of TpDb solution. d) Cytotoxicity test of TpDb; which illustrate the cell viability with different concentrations of TpDb. e–h) Cell staining results where the green and red one indicate the living and dead cells, respectively, e) shows control, i.e., without TpDb f) with 0.5 µg mL^−1^ concentration of TpDb, g) with 5 µg mL^−1^ concentration of TpDb h) with 25 µg mL^−1^ concentration of TpDb.

The schematic representation of the synthesis TpDb is shown in **Figure** **1**a. The 1, 4‐diacetylbenzene (Db) was initially mixed with PTSA (para‐toluene sulphonic acid) and then reacted with 1, 3, 5‐ triformylphloroglucinol (Tp) resulting in an aldol condensation reaction. The polymerization reaction yields a conjugated enone (*─*C═C*─*C═O) framework along with redox‐active triol (*─*OH) at the benzene core. Figure 1b shows a scanning electron microscope image (SEM) of the TpDb depicting a high density of flakes. Figure 1c represents the schematic diagram of the preparation of TpDb solution starting from its flakes. The cytotoxicity test of TpDb, illustrated in Figure 1d, shows the cell viability with different concentrations, ranging from 0 to 250 µg mL^−1^. The *y*‐axis represents the cell viability, where higher values indicate greater numbers of live cells. In the experiment, the cell viability maintained a relatively high level with different concentrations of TpDb. It shows over 70% cell viability for 50, 100, and 250 µg mL^−1^ TpDb concentration, which indicates it does not induce significant cellular toxicity or promote widespread cell death. This evidence supports the material biocompatibility of TpDb, which highlights its green property and the potential for biomedical applications. Figure 1e–h depicts the staining patterns of control (without TpDb) and with various concentrations of TpDb. The green and red colors represent living and dead cells, respectively. Figure 1e depicts a population of exclusively green‐stained cells, indicative of a healthy and viable control group. Furthermore, Figure 1f–h demonstrates predominantly green‐stained cells, suggesting sustained cell viability upon increasing TpDb concentrations to 0.5, 5, and 25 µg mL^−1^, respectively. These staining results collectively indicate that TpDb does not induce widespread cell death, supporting its biocompatibility. The Fourier‐transform infrared (FT‐IR) spectroscopy showed stretching vibration at 1610 and 1683 cm^−1^ corresponding to C═C and ketone C═O bonds which suggest the formation of a conjugated enone in the framework upon the dehydration process (Figure S3, Supporting Information). Furthermore, the ^13^C solid‐state NMR spectroscopy validated the presence of the C═O from the enone which appeared at ≈192 ppm (Figure S4, Supporting Information). Additionally, distinctive signals for the olefin C═C and the C*─*O groups were detected at 149 and 161 ppm, respectively.

**Figure 2:**
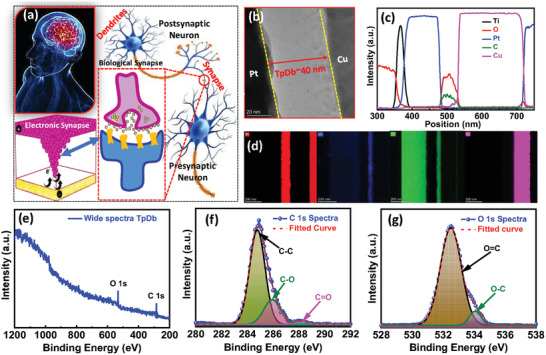
a) Schematic illustration of the biological synapse and the equivalent artificial/electronic synapse Cu/TpDb/Pt memristor device. b) The cross‐section TEM images of the devices with the scale bar of 20 nm. c) EDS elemental line profile to validate the various elements in the device. d) EDS mapping of various elements Pt, C, O, and Cu presented in the Cu/TpDb/Pt memristive device. e) Wide scan spectra X‐ray photo spectroscopy of TpDb/Pt. f) C 1s spectra of X‐ray photo spectroscopy of TpDb/Pt. g) O 1s spectra of X‐ray photo spectroscopy of TpDb/Pt.

**Figure** **2**a depicts a schematic illustration of the biological synapse in humans and the equivalent electronic/artificial synapse based on the Cu/TpDb/Pt memristive device. The human brain, shown at the top left corner, stores information and is responsible for the learning process.^[^ ^19^ ^]^ In the biological synapse, a presynaptic neuron is responsible for transferring Ca^2+^ or Na^2+^ ions, i.e., neurotransmitters, to the postsynaptic neurons when the input impulses are received, and thus, the biological synapse weight changes via synaptic plasticity.^[^ ^20^ ^]^ Similarly, the demonstrated device in this work, based on the organic 2D polymer, TpDb, mimics an artificial synapse where the migration of Cu ions under a certain applied voltage impulse results in the modulation of its conductance. An electronic or artificial synapse is also shown in the same figure, where Cu acts as presynaptic neuron, while Pt is the postsynaptic neuron and TpDb represents the active synapse layer where the signal transmission would occur. The continuous change in the resistance of the device is equivalent to the synaptic change and is modulated by supplying continuous electrical spikes.^[^ ^21^ ^]^ Figure 2b represents the cross‐sectional HRTEM image of the Cu/TpDb/Pt synaptic structure on the scale bar of 20 nm, which shows a continuous film of the TpDb sandwiched in between the electrodes. Figure 2c depicts the energy dispersive spectroscopy (EDS) line profile of the synaptic device. The EDS line profile confirms the multilayer structure of the device and validates the presence of platinum (Pt), carbon (C), oxygen (O) (C and O because of organic polymer active sample TpDb), and copper (Cu). Figure 2d shows the elemental mapping of Cu, C, Pt, O, and Ti which also validates the presence of each element in the device.

The successful drop‐casted layered structure of TpDb was verified by XPS survey which led us to a deeper understanding of bonding (see Figure 2e–g). Figure 2e shows the presence of C and O elements that were found in the wide XPS profile. Carbon 1s spectra consisted of three peaks concentrated at different binding energies 284.3, 286.0, and 288.2 eV attributed to C═C, C*─*O, and C═O (Figure 2f). The two typical O 1s signals concentrated at about 532.4 and 534.1 eV can be assigned to O═C and O*─*C, respectively (Figure 2g).

**Figure 3:**
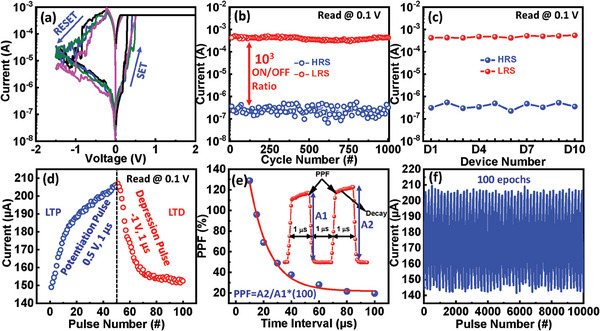
Design and electrical characterization. a) *I*–*V* characteristics of the TpDb‐based device for 1st, 100th, 500th, and 1000th cycle. b) DC endurance or (LRS/HRS current) of the TpDb device. c) Device to device uniformity of D1, D4, D7, and D10 devices. d) Synaptic behavior of long‐term potentiation (LTP) and depression (LTD) of the TpDb device by 50 SET pulses and 50 RESET pulses. e) The PPF index of the device and Voltage pulse scheme for Paired pulse Facilitation (PPF) of the synaptic device (inset). f) Repeatability of LTP and LTD characteristics of the device with total number of 100 epochs.

Using the synthesized material, a Cu/TpDb/Pt device is fabricated as described in the Experimental Section. The resulting device is next electrically characterized. All of the electrical characterizations were done with the voltage applied to the top electrode (Cu), while the bottom electrode (Pt) was grounded. A forming voltage was found at ≈2.5 V to trigger the transfer from the high resistance state (HRS) to the low resistance state (LRS). **Figure** **3**a,b illustrates the current‐voltage (*I–V)* characteristics and direct current (DC) endurance for Cu/TpDb/Pt device. After forming, the device changes its resistance state from LRS to HRS (called RESET or depression process) with a negative sweep of 0 to −1.5 V and HRS to LRS (SET or potentiation process) with a positive sweep of 0–2 V as shown in Figure 3a. The device shows good endurance of 1000 DC cycles with a large LRS/HRS ratio of about 10^3^ as shown in Figure 3b. The switching ratio is defined as the ratio between the currents in the high and low resistance states of the memristor. High switching ratio allows the accurately map the network weight value to device conductance. Ideally, the lowest conductance state (OFF‐state) should be low enough to represent the zero weight in the algorithm, making the dynamic range (conductance ON/OFF ratio) sufficiently large. In reality, the ON/OFF ratio is always finite and normally not large enough. The high switching ratio of our device enables the hardware synapse to accurately map the neural network weights, which suits for the neuromorphic applications. Figure 3c shows the device‐to‐device uniformity of randomly chosen 10 devices, and the highly stable LRS and HRS states confirm the high reproducibility of the device. Figure 3d exhibits current changes in the device by applying 50 potentiation (P) pulses and 50 depression (D) pulses. The current of the synaptic device can be varied with the number of provided P and D pulses. Paired‐pulse facilitation (PPF) is a crucial biological feature of the synaptic devices which is depicted in Figure 3e.^[^ ^22^, ^23^ ^]^ More specifically, in Figure 3e (inset) a voltage pulse scheme that is applied to calculate the PPF is represented. 3e The Cu/TpDb/Pt device achieved the PPF characteristic where the current was further increased by the second pulse compared to the first. After supplying a 0.8 V presynaptic voltage for Δ*t* = 1 µs, the change in the current is represented as A_1_. Next, after applying a 0.8 V postsynaptic voltage for Δ*t* = 1 µs, the obtained increment in the current (A_2_) in the neuron is known as the excitatory postsynaptic current (EPSC).^[^ ^24^ ^]^ Thus, two continuous electrical pulses were applied to attain the PPF index by using (A_2_‐A_1_/A_1_)*100, which showed a stable and gradual decrease in the PPF index (as shown in Figure 3e). Figure 3f depicts stable potentiation and depression cycles for at least 10 000 electrical applied pulses. Such characteristics of the memristive synaptic device confirm its ability to mimic the P and D functions of the biological brain. Thus, the Cu/TpDb/Pt device exhibits promising electrical characteristics and is promising for neuromorphic computing.

**Figure 4:**
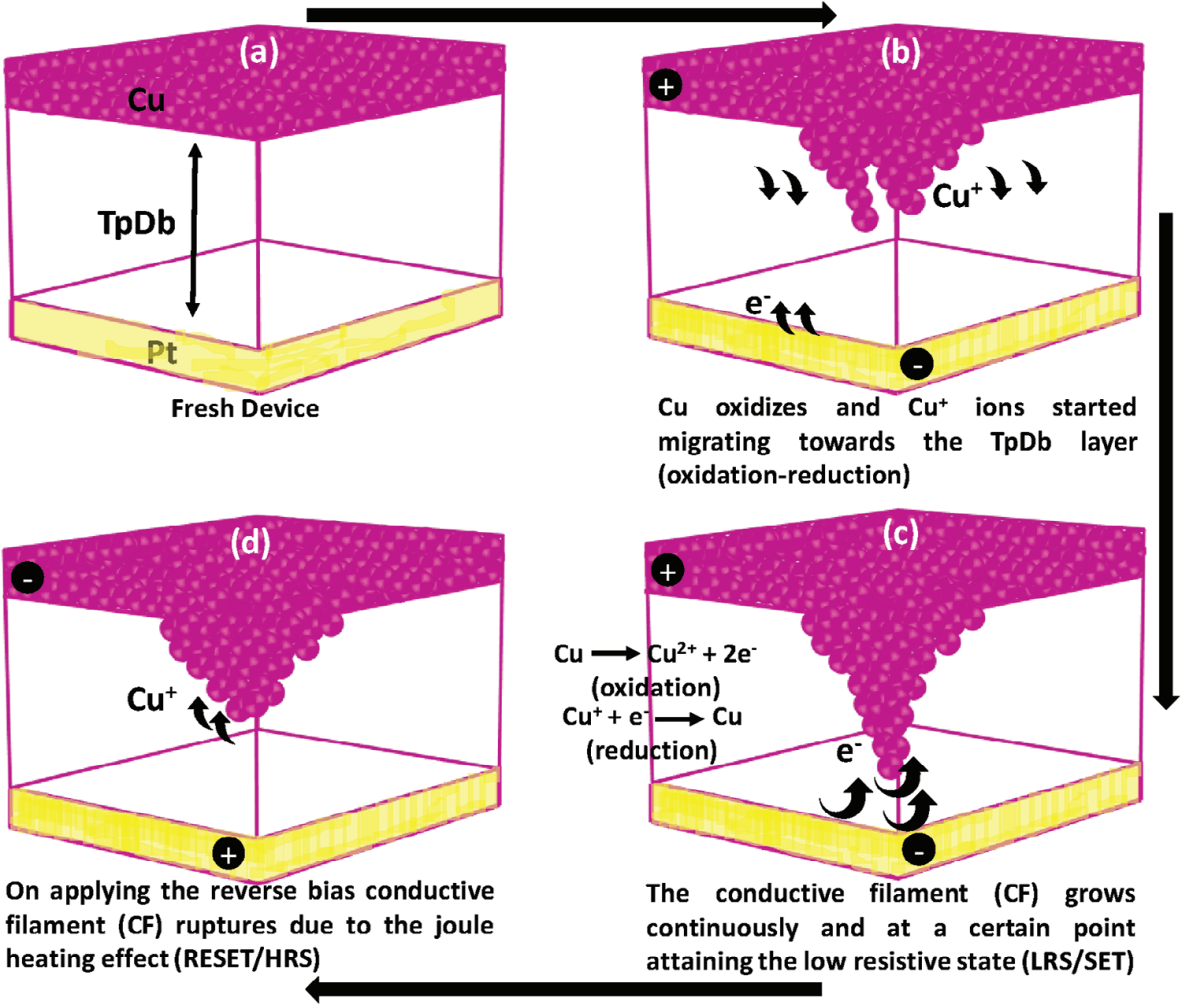
Illustration of the electrochemical processes during resistive switching. a) Schematic of the fresh device. b) When positive (+) bias is applied on the top electrode, Cu starts oxidizing and Cu+ ions start moving toward TpDb layer. c) Migration of Cu+ ions in the TpDb layer indicating oxidation and reduction reactions happening before Cu+ reaches to the bottom electrode. d) The conductive filament (CF) grows continuously and at a certain point, the device attains the low resistance state (LRS) which implies the SET process. e) By applying the reverse bias, the conductive filament ruptures due to the joule heating effect (HRS/RESET).

To better understand the reported results, a mechanism for the conductive filament (CF) formation and rupture is proposed which is the main cause of the resistive switching behavior (**Figure** **4**). Figure 4a shows the original device structure (before applying any bias). When a positive bias is applied on the top electrode, Cu atoms get oxidized into Cu^+^ ions and start diffusing into the TpDb layer (see Figure 4b). By further increasing the voltage, the oxidation and reduction processes, shown in Figure 4c, result in full conductive filament growth. This stage of full growth of CF is also known as SET or LRS. When a negative bias is applied as shown in Figure 4d, the CF ruptures due to the Joule heating effect which means that the device is switched to the RESET stage or the HRS.^[^ ^25^, ^26^, ^27^, ^28^, ^29^ ^]^

**Figure 5:**
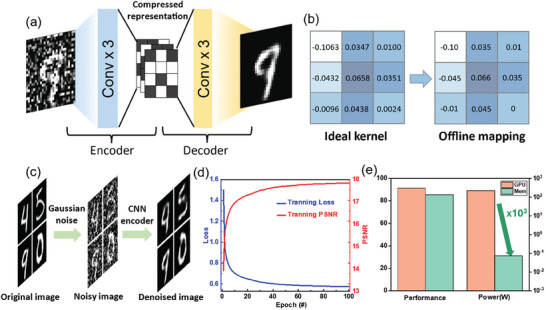
a) Network structure of CNN autoencoder b) The kernels (left) are obtained from the ideal software test. The offline mapping kernels (right) are mapping from the corresponding ideal kernel values with device conductance. c) The figure visualization for different steps. d) The training loss and PSNR result with the training epochs. e) The comparison result between GPU and our memristor device.

Next, the application of the TpDb memristor is demonstrated in two AI‐based applications: noise reduction and edge detection. In the first application, a convolutional neural network (CNN) autoencoder is used to reduce the image noise, where TpDb memristor is employed as the convolutional kernel. The network architecture is shown in **Figure** **5**a which consists of an encoder and decoder. Each encoder or decoder contains three 3×3 convolutional blocks followed by the max pooling function. For the training data, we manually add Gaussian noise to originally clean images in MNIST dataset as an external noisy perturbation, while the origin images are utilized as a training label. During the training, the CNN autoencoder is guided to denoise the images by learning to remove unwanted noise while preserving the important underlying features.^[^ ^30^ ^]^ As shown in Figure 5b, after the offline training process, each convolutional kernel values are mapped to hardware device conductance for in‐memory computations. The results are presented in Figure 5c, where TpDb‐based CNN encoder is shown to significantly help in reducing the noise level of image. The training loss and peak signal‐to‐noise ratio (PSNR) are used to evaluate the network performance, as shown in Figure 5d. Figure 5e shows the comparison of performance and power consumption between our memristor and the Tesla P40 graphics processing unit (GPU). The GPU and memristor based platform exhibit comparable performance, as assessed by the normalized value of PSNR and the mean square error (MSE). However, in terms of energy efficiency, we evaluate the power consumption through the traditional GPU and memristor device array.^[^ ^31^ ^]^ Our device demonstrates a significant advantage, consuming 10^3^ times less energy compared to the Tesla P40 GPU, thus contributing towards more sustainable systems. The evaluation of the power and energy cost is explained in Note‐S10 (Supporting Information). The experimental results demonstrate the good programming and learning abilities of the TpDb device and its potential to work as a convolutional kernel and perform in‐memory computing tasks for noise reduction.

**Figure 6:**
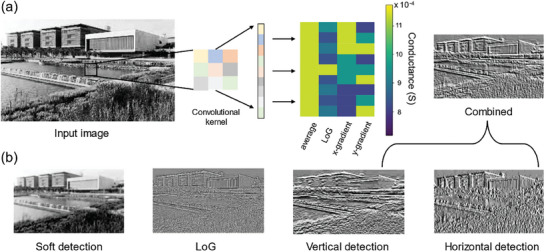
a) The procedure of hardware‐based convolutional image processing performed on the KAUST campus image. b) Different convolutional operators can help extract features of the image for different detection applications.

The second application in edge detection is described next. Convolutional operations are a widely used method based on MAC (multiply and accumulate) operations to extract image features. Different convolutional operators can be adopted for different image‐processing tasks. For example, the Laplacian of Gaussian (LoG) operator can be useful for detecting blobs and smoothing images, while Sobel operator is suitable for edge detection.^[^ ^32^ ^]^ **Figure** **6**a presents the procedure of the hardware‐based convolutional image processing performed on the KAUST campus image. The pixel values of the original image can be mapped to the input voltage signals. To extract the feature of the image, convolution operations are performed and traversed between neighboring pixels. Specifically, the convolutional kernels are directly used and programmed using the TpDb device. The convolution operation is a typical multiply and accumulate operation, where each pixel is multiplied by a specific convolutional kernel, and the resulting products in each column are then added together arithmetically. Finally, the output current map is measured and then plotted as the output processed image. As shown in Figure 6b, different convolutional operators can help extract features of the image for different detection applications. The combined one contains the information for both vertical and horizontal edges. The well‐presented image features confirm the effectiveness of feature extraction through the programmed device's convolutional filter.

**Figure 7:**
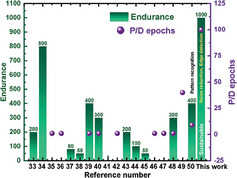
Comparison of our TpDb memristor with previously reported organic and polymeric‐based memristor devices.

To benchmark the TpDb device performance, **Figure** **7** shows a fine comparison of resistive switching characteristics such as endurance (left *y*‐axis) and memristive synaptic properties, such as P/D epochs (right *y*‐axis) between previously reported organic polymer‐based devices and this work ^[^ ^33^, ^34^, ^35^, ^36^, ^37^, ^38^, ^39^, ^40^, ^41^, ^42^, ^43^, ^44^, ^45^, ^46^, ^47^, ^48^, ^49^ ^]^. Our device shows the best reliability in terms of endurance (up to 1000 cycles) and number of P/D epochs. Moreover, while the pattern recognition application has been previously reported,^[^ ^50^ ^]^ our device is more suitable for noise reduction and edge detection applications. It is also worth noting that the majority of previously reported devices are not environmentally friendly because they use nonrecyclable or toxic materials for instance. Table S1 (Supporting Information) shows a more detailed comparison between the resistive switching characteristics of the demonstrated Cu/TpDb/Pt device in this work and previously reported organic and polymer‐based devices. Moreover, the TpDb‐based memristor shows superior and unique performance in terms of endurance, P/D epochs, application, and most importantly its contribution to lower e‐waste and thus, sustainability.

## 3 Conclusion

In conclusion, in this work, a memristive device using a green‐synthesized carbonyl‐rich organic 2D‐polymer (TpDb), sandwiched between electrodes that can be potentially fully recycled, is demonstrated for application in two AI‐based tasks: noise reduction and edge detection. The device showed an endurance of 1000 DC cycles with a large LRS/HRS ratio of about 10^3^. The good device‐to‐device uniformity and highly stable LRS and HRS states confirm the high reproducibility of the devices. The devices also showed synaptic properties including LTP, STP, and PPF. Using the obtained characteristics, experiments are conducted to evaluate the performance of a TpDb‐based convolutional kernel for application in edge detection and image de‐noising tasks. The efficient matrix computation operation using the TpDb device opens a promising route for emerging applications in the hardware accelerators to boost AI and machine learning algorithms sustainably.

## 4 Experimental Section

### Green Solid‐State Synthesis of TpDb Flakes

First, 170 mg of Db was mixed with 453 mg of PTSA for the production of the corresponding enol. The mixture of Db‐PTSA was collected and 100 mg of Tp was mechano‐mixed with the above mixture resulting in a white color paste. The white color paste was then thermally treated for 24 h at 90 °C. After that, black‐colored flakes were collected and washed with *N*, *N*‐dimethylacetamide (at 90 °C), and subsequently with water (at 60 °C) and acetone. In the final step, the collected flakes were dried and used further as shown below.

### Preparation of TpDb Solution Based on the As‐Synthesized TpDb Flakes

A mechanical grinding strategy was opted to prepare the TpDb sample. A small piece of as‐synthesized TpDb bulk sample was placed in a mortar and pestle. Then 2–3 drops of methanol were added to ground the flakes using a pestle at room temperature for 30 min. The fine powder collected after 30 min of grinding was then dispersed in isopropanol (IPA) and stirred for 15 h at 500 rpm. The TpDb solution was obtained thereafter.

### Cytotoxicity test:—Cell Culture

HeLa cells, derived from a human cervical cancer cell line, were cultured in Dulbecco’s Modified Eagle’s Medium (DMEM): (Solarbio, 31 800). This medium was supplemented with 10 wt % fetal bovine serum (FBS): (BI, C04001), 100 units mL^−1^ penicillin, and 100 mg mL^−1^ streptomycin (NCM Biotech, C100C5). The cells were cultured in an incubator at 37 °C and 5% CO_2_.

### Cytotoxicity Test—Cell Cytotoxicity Assay

Cytotoxicity of TpDb to the HeLa cells was measured by the CCK8 method. HeLa cells at a concentration of 1 × 104 cells per well were seeded in DMEM complete medium into microplates (tissue culture grade, 96 wells, flat bottom). After 24 h incubation, the medium was replaced with fresh DMEM with different concentrations of TpDb. Then the cell viability was assayed based on the colorimetric CCK8 cell proliferation assay kit (Merck,96 992) according to the manufacturer's protocol.

### Cytotoxicity Test—Dead/Live Cell Staining

The cells were seeded into a 24‐well plate with different treatments, and then stained by LIVE/DEAD Viability/Cytotoxicity Kit following manufacturer's instructions (Thermofisher, L32250). Both fluorescent and bright‐field images were obtained by an inverted fluorescent microscope. The dead and live cells in different pictures were automatically counted by ImageJ software, and then the dead cell proportion was calculated.

### Fabrication of TpDb Memristor

P+−Si substrate was used for device fabrication. First, the native oxide was etched using buffered oxide etchant for 3 min and the Si wafer was then rinsed with deionized (DI) water. A nitrogen gun was used to dry the wafer. Next, an isolation SiO_2_ layer was deposited by plasma‐enhanced chemical vapor deposition (PECVD) at 350 °C. A 30 nm thick Titanium (Ti) layer was then deposited as adhesion layer using sputtering. Then, a 100 nm thick layer of Platinum (pt) was deposited by sputtering as the bottom electrode. In the next step, TpDb solution was drop‐casted on the Ti layer and a 40 nm thick film was deposited. Finally, a 200 nm Cu film was deposited by RF sputtering under pure Ar ambiance using a metal shadow mask (100 µm in dia). Following these steps, the Cu/TpDb/Pt memristive device was fabricated.

### Structural and Electrical Characterization of the Devices

High‐resolution transmission electron microscopy (HRTEM‐ JEOL JEM‐2010F) was employed to study the device's cross‐section and nanolayers. The X‐ray photoelectron spectroscopy (XPS) was performed on the TpDb sample in a high vacuum using a Kratos Amicus XPS equipment with a monochromatic Al Kα X‐ray source operating at 10 kV. The electrical measurement was done using the experimental setup Keysight B1500 A semiconductor device analyzer and a tunable light source of Newport Corporation.

## Conflict of Interest

The authors declare no conflict of interest.

## Supporting information



Supporting Information

## Data Availability

The data that support the findings of this study are available from the corresponding author upon reasonable request.
