# Supplementary material for: Energy Efficient Memristor Based on Green‐Synthesized 2D Carbonyl‐Decorated Organic Polymer and Application in Image Denoising and Edge Detection: Toward Sustainable AI
Source: Adv Sci (Weinh). 2024 Sep 9;11(45):2408648. doi: 10.1002/advs.202408648 (PMC11615820; doi:10.1002/advs.202408648)
Supplement: Supplementary file 1 — Supporting Information [file ADVS-11-2408648-s001.docx]

**Energy Efficient Memristor Based on Green-Synthesized Two-Dimensional Carbonyl-Decorated Organic Polymer and Application in Image Denoising and Edge Detection: Towards Sustainable AI**

Pratibha Pal^#^, Hanrui Li^#^, Ruba Al-Ajeil, Abdul Khayum Mohammed, Ayman Rezk, Georgian Melinte, Ammar Nayfeh, Dinesh Shetty*, Nazek El-Atab*

Dr. P. Pal, H. Li, Prof. N. El-Atab

Smart, Advanced Memory Devices and Applications (SAMA) Laboratory, Electrical and Computer Engineering Program, Computer Electrical Mathematical Science and Engineering Division, King Abdullah University of Science and Technology (KAUST), 23955 Thuwal, Kingdom of Saudi Arabia. Email: [nazek.elatab@kaust.edu.sa](mailto:nazek.elatab@kaust.edu.sa)

R. Al-Ajeil, Dr. A. K. Mohammed, and Prof. D. Shetty
Department of Chemistry, Khalifa University of Science & Technology, PO Box: 127788, Abu Dhabi, United Arab Emirates. E-mail: [dinesh.shetty@ku.ac.ae](mailto:dinesh.shetty@ku.ac.ae)

Prof. D. Shetty

Center for Catalysis & Separations (CeCaS), Khalifa University of Science & Technology, P.O. Box. 127788, Abu Dhabi, UAE.

Dr. G. Melinte

Core Labs, King Abdullah University of Science and Technology, Thuwal, 23955-6900 Saudi Arabia

Dr. A. Rezk, Prof A. Nayfeh

Department of Electrical Engineering, Khalifa University of Science & Technology, Abu Dhabi 127788, UAE


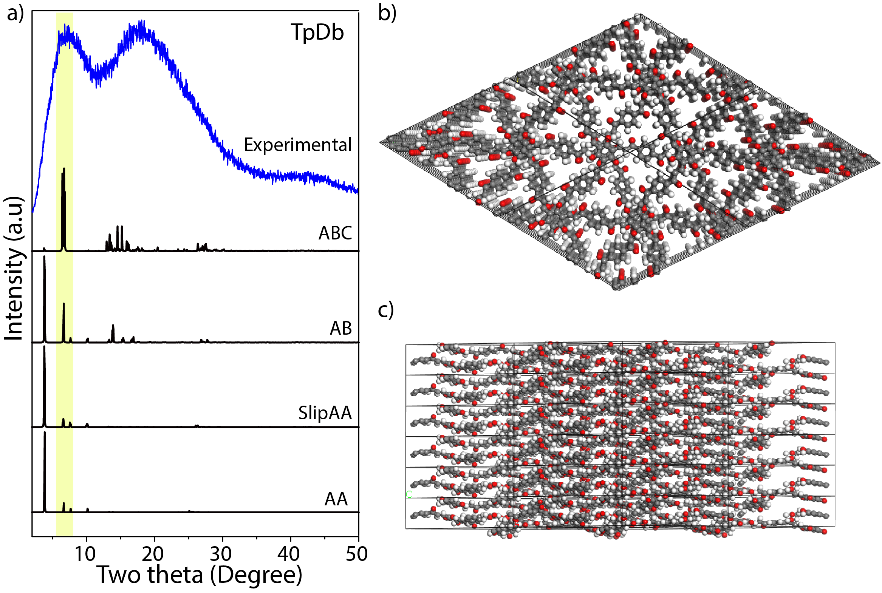


Figure S1: a) The PXRD profile of TpDb with simulates PXRDs of various stacking models; b) The theoretical structure ABC stacked of TpDb (horizontal view) ; c) The theoretical structure ABC stacked of TpDb (vertical view)


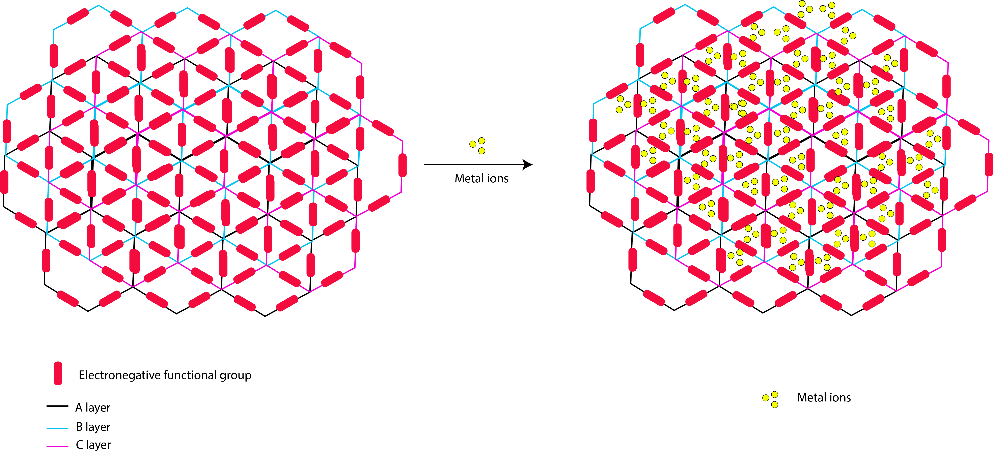


Figure S2: The graphical representation of metal ions in the channels of ABC-stacked layers with electronegative functional groups. The columnar stacking of these channels further provide an efficient metallization process upon voltage variation.


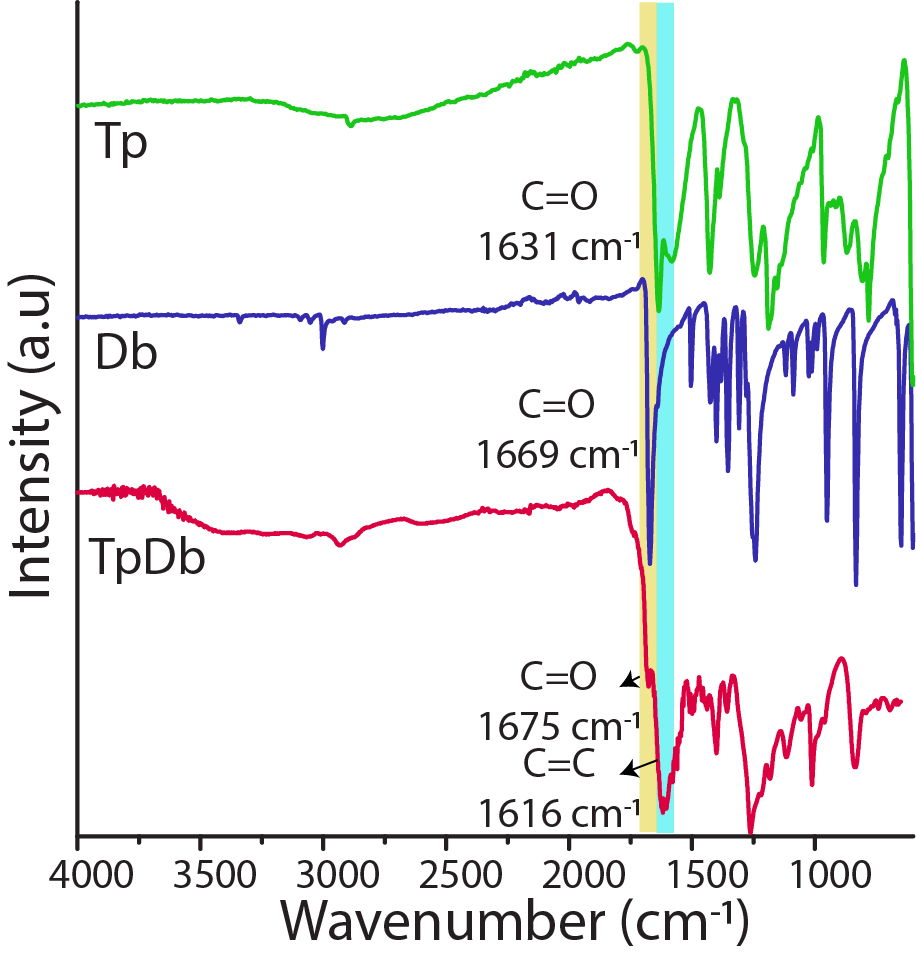


Figure S3: The FT-IR spectrum of TpDb and monomers (Tp and Db)


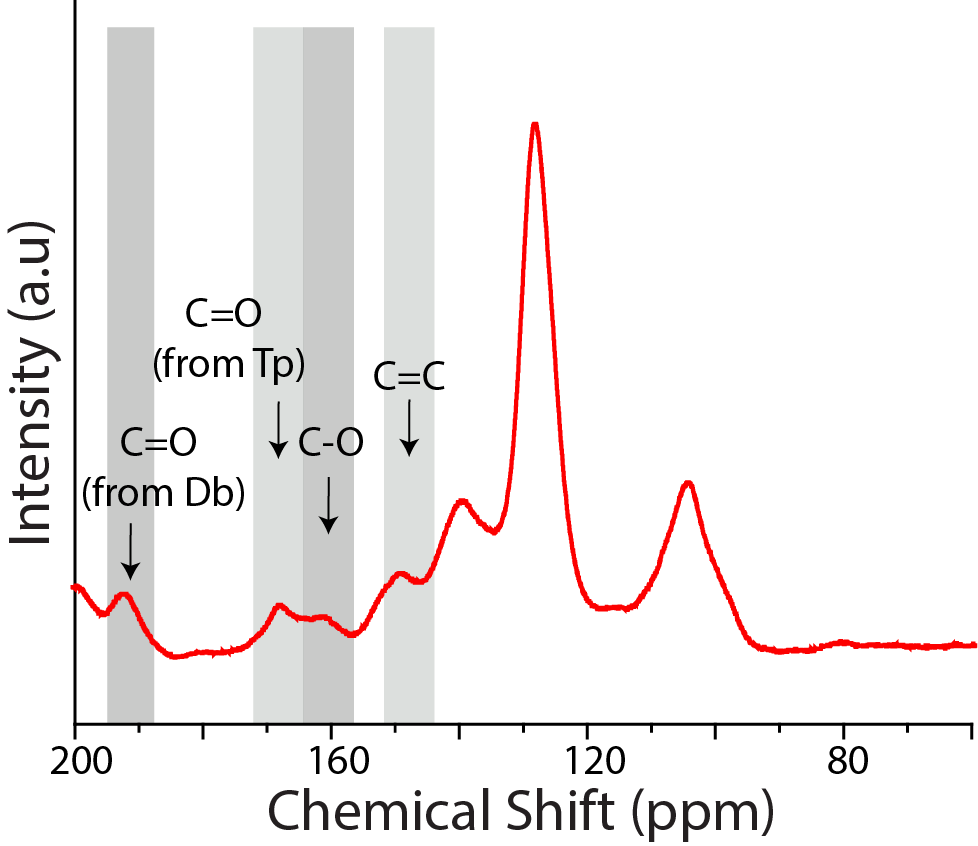


Figure S4: The 13C CP MAS solid state NMR spectrum of TpDb

Figure S5: Schematic diagram of the fabrication process flow of Cu/TpDb/Pt memristor device

A P^+^-Si as a substrate was etched by BOE (buffer oxide etchant) for 3 minutes. After that, Si was rinsed by DI (deionized) water and dried using a nitrogen gun. In the next step, silicon oxide (SiO_2_) layer was deposited by plasma enhanced chemical vapor deposition (PECVD) at 350^o^C. Then, an adhesion layer Titanium (Ti) was sputtered at the top of SiO_2_. A bottom electrode Platinum (Pt) of 100-nm thickness was deposited by using the sputtering tool. TpDb solution was then drop-casted on the top surface of the bottom electrode at 80^o^C. As the final step, a top electrode Copper (Cu) was deposited by RF sputtering using the metal shadow mask (100 μm). A new structure of Cu/TpDb/Pt memristive device was thus fabricated by following the above steps.

Figure S6: Cumulative probability curve for cycle-to-cycle variability of Cu/TpDb/Pt memristor device

In figure S6, the cycle-to-cycle variability was studied for the Cu/TpDb/Pt memristor device using 100 continuous cycles. The device shows highly stable LRS/HRS without degradation for cumulative probability.

Figure S7: Retention states of LRS/HRS for Cu/TpDb/Pt device

The reliability of the Cu/TpDb/Pt memristor device was investigated by measuring the retention properties of LRS and HRS. Figure S7, depicts the high stability of both LRS and HRS states. The memristor device has stable states of LRS and HRS for more than 10^5^ seconds without any degradation.

Figure S8: Pulse schemes applied to the Cu/TpDb/Pt synaptic memristor for achieving potentiation and depression behaviors

$$G_{LTP}=B* \left[ 1-\exp\left( -\frac{P}{A} \right)+G_{min} \right]\ldots\ldots\ldots\ldots\ldots\ldots1$$

$$G_{LTD}= -B*\left[ 1-\exp\left( \frac{P-P_{max}}{A} \right) \right]+G_{max}\ldots\ldots\ldots\ldots\ldots\ldots2$$

$$B=\frac{G_{max}- G_{min}}{1-exp(-\frac{P_{max}}{A})}\ldots\ldots\ldots\ldots\ldots\ldots3$$


Figure S9: (a) The ANN network structure contains input layer, hidden layer and output layer (b) The recognition accuracy increases for the TpDb memristive device with the training epochs (c) Confusion matrix with (i) Initial training state (ii) after 25 epochs (iii) after 50 epochs

Figure S 9a shows the artificial neural network (ANN) implemented using potentiation/depression data of electrical training epochs. The Modified National Institute of Standards and Technology (MNIST) database is being used for recognition purposes in ANN architecture. The ANN system consists of three layers with 28x28 input neurons, 100 hidden neurons and 10 output neurons as shown in figure S 9a. The accuracy results are shown in figure S 9b. After 150 epochs the synaptic device’s accuracy got stable at approximate 94% slightly lower than the software’s accuracy (96%). Figure S 9c (i), (ii) and (iii) represent the confusion matrix for the initial state, after training 25 epochs and after 50 epochs respectively. The diagonal diamond shaped colored line signifies the improvement in the recognition ability of the electrical synapse.

**Note-10: Power evaluation**

The power consumption (P) of the memristor is evaluated using the following formula [19]:

$$P=UI$$

where U is the applied voltage pulse amplitude, I represent the average current generated from the voltage pulse out of the series voltage pulses (amplitude of 0.5 V). By multiplying it by the simulated array size of 32 × 32, the power consumption of the memristor is calculated to be 9.2 × 10^−2^ W. The power consumption of GPU was evaluated through TDP and GPU utilization. Specifically, the Tesla P40's TDP of 250W, operating at GPU utilization of 74%, results in a power consumption of 185W.

*Table S1: Comparison between the resistive switching characteristics of organic and polymeric based memristors and application in neuromorphic computing.*

**References**

1. J. Liu, F. Yang, L. Cao, B. Li, K. Yuan, S. Lei, W. Hu, *Adv. Mater.* 2019, 31, 1902264 (<https://doi.org/10.1002/adma.201902264>).
2. T. Zhang, D. Guerin, F. Alibart, D. Vuillaume, K. Lmimouni, S. Lenfant, A. Yassin, M. Ocafrain, P. Blanchard, and J. Roncali, *J. Phys. Chem. C.* 2017, 121, 18, 10131–10139 (<https://doi.org/10.1021/acs.jpcc.7b00056>).
3. S. Li, F. Zeng, C. Chen, H. Liu, G. Tang, S. Gao, C. Song, Y. Lin, F. Pan and D. Guo, *J. Mater. Chem. C*. 2013, 1, 5292-5298 (<https://doi.org/10.1039/C3TC30575A>).
4. F. Zeng, S. Li, J. Yang, F. Pan and D. Guo, *RSC Adv.* 2014, 4, 14822-14828 (<https://doi.org/10.1039/C3RA46679E>).
5. Y.-C Lai, Y.-X. Wang, Y.-C. Huang, T. -Y. Lin, Y.-P. Hsieh, Y.-J. Yang, Y.-F. Chen, *Adv. Funct. Mater.* 2014, 24: 1430-1438 (<https://doi.org/10.1002/adfm.201302246>).
6. Y. Lin, X. Zhang, X. Shan, T. Zeng, X. Zhao,Z. Wang, Z. Kang, H. Xu and Y. Liu, *J. Mater. Chem. C.* 2020, 8, 14789-14795 (<https://doi.org/10.1039/D0TC03907A>).
7. T. Hussain, H. Abbas, C. Youn, H. Lee, T. Boynazarov, B. Ku, Y.-R. Jeon, H. Han, J. H. Lee,C. Choi, T. Choi, *Adv. Mater. Technol.* 2022, 7, 2100744 (<https://doi.org/10.1002/admt.202100744>).
8. Z. Zhao, M. E. El-Khouly, Q. Che, F. Sun, B. Zhang, H. He, Y. Chen, *Angew. Chem. Int. Ed.* 2023, 62, e202217249; *Angew. Chem.* 2023, 135, e202217249 (<https://doi.org/10.1002/anie.202217249>).
9. S.-H. Lee, H. -L. Park, C. -M. Keum, I. -H. Lee, M. -H. Kim, S. -D. Lee, *Phys. Status Solidi RRL*. 13: 1900044 (<https://doi.org/10.1002/pssr.201900044>).
10. G. Liu, C. Wang, W. Zhang, L. Pan, C. Zhang, X. Yang, F. Fan, Y. Chen, R. - W. Li, *Adv. Electron. Mater.* 2: 1500298 (<https://doi.org/10.1002/aelm.201500298>).
11. Z. Jin, G. Liu, J. Wang, *AIP Advances*. 2013, 3, 052113 (<https://doi.org/10.1063/1.4804948>).
12. S. P. Park, Y. J. Tak, H. J. Kim, J. H. Lee, H. Yoo, H. J. Kim, *Adv. Mater.* 2018, 30, 1800722 (<https://doi.org/10.1002/adma.201800722>).
13. M.-K. Kim and J.-S. Lee, *ACS Appl. Mater. Interfaces.* 2018, 10, 12, 10280–10286 (<https://doi.org/10.1021/acsami.8b01781>).
14. C. D. P.-Socorro, S. G.-Santamarina, L. Mardegan, L. E.-Moreno, H. J. Bolink, S. C.-Serra, E. Coronado, *Adv. Electron. Mater.* 2022, 8, 2101192 (<https://doi.org/10.1002/aelm.202101192>).
15. X. Yang, C. Wang, J. Shang, C. Zhang, H. Tan, X. Yi, L. Pan, W. Zhang, F. Fan, Y. Liu, Y. Chen, G. Liu and R.-W. Li, *RSC Adv.* 2016, 6, 25179-25184 (<https://doi.org/10.1039/C6RA02915A>).
16. H.-L.Park, M.-H. Kim, M.-H. Kim and S.-H. Lee, *Nanoscale.* 2020, 12, 22502-22510 (<https://doi.org/10.1039/D0NR06964G>).
17. X. Luo, J. Ming, J. Gao, J. Zhuang, J Fu, Z. Ren, H. Ling, L. Xie, *Front. Neurosci.* 2022, 16:1016026 (<https://doi.org/10.3389/fnins.2022.1016026>).
18. M.-H. Kim, H. -L. Park, M. -H. Kim, J. Jang, J. -H. Bae, I. M. Kang and S.-H. Lee, *npj Flex Electron.* 2021, 5, 34 (https://doi.org/10.1038/s41528-021-00132-w).
19. Li X, Zhong Y, Chen H, et al. *Adv. Mater.* 2023, 35(37): 2203684 (<https://doi.org/10.1002/adma.202203684>).
